# A Case Report and Surgical Video Presentation of Totally Laparoscopic Right Trisectionectomy: Addressing the Challenges of Hepatolithiasis

**DOI:** 10.7759/cureus.97662

**Published:** 2025-11-24

**Authors:** Camila Sotomayor, Cristobal Vildosola, Alan Camhi, Jose M González, Javiera Torres, Eduardo Briceño

**Affiliations:** 1 Department of Hepatobiliary and Pancreatic Surgery, Pontificia Universidad Católica de Chile, Santiago, CHL; 2 Department of Pathology, Pontificia Universidad Católica de Chile, Santiago, CHL

**Keywords:** hepatolithiasis, intrahepatic lithiasis, laparoscopic hepatectomy, major hepatectomy, minimally invasive hepatectomy

## Abstract

Hepatolithiasis, the presence of calculi within the intrahepatic bile ducts, is a rare but challenging condition that can lead to complications such as cholangitis and liver dysfunction. We present the case of a 65-year-old male patient who presented with a history of abdominal pain and chills without clinical jaundice or fever. Laboratory tests revealed mild abnormalities in liver function tests with a cholestatic pattern. Magnetic resonance cholangiopancreatography (MRCP) revealed cholelithiasis, choledocholithiasis, and hepatolithiasis in segments V and VIII. He was started on antibiotic therapy with ciprofloxacin, and an ERCP and cholangioscopy were performed for the extraction of the choledocholithiasis. During the procedure, a stenosis of the right hepatic duct was found and biopsied. In addition, papillotomy was performed with the extraction of multiple stones and biliary sludge, and a 10 Fr plastic stent was placed in the right duct. The biopsy result revealed inflammatory tissue, with neoplasia not completely excluded. An elective laparoscopic right hepatectomy was scheduled. During surgery, ultrasound and cholangiography revealed the presence of hepatolithiasis in segment 4, prompting the decision to change the surgical technique to an extended right hepatectomy, which was performed entirely laparoscopically without incidents. Histopathological analysis of the biliary margin confirmed the absence of malignancy. Totally laparoscopic extended right hepatectomy is a feasible and effective treatment for hepatolithiasis, providing good outcomes with minimal morbidity. This case underscores the importance of surgical intervention in managing complex hepatobiliary disorders.

## Introduction

Hepatolithiasis refers to the presence of stones within the bile ducts inside the liver. This condition can lead to serious complications, including recurrent episodes of cholangitis, narrowing of the bile ducts (strictures), localized liver atrophy, and even cholangiocarcinoma (CCA) [1].

Hepatolithiasis is a multifactorial disease with a complex and not yet fully understood pathophysiology. Contributing factors include cholestasis, biliary infection, anatomical abnormalities, altered bile metabolism, diet, and genetic mutations. Strictures and deformities in the biliary tree disrupt bile flow and favor crystal formation, while dysfunction of the sphincter of Oddi and biliary dysbiosis further promote inflammation and stone development. Parasitic infections and low-fat, low-protein diets also contribute to bile stasis. These mechanisms often interact, creating a vicious cycle of biliary injury, fibrosis, and recurrent stone formation [2].

Liver resection is considered the treatment of choice for hepatolithiasis, particularly in patients with localized disease, recurrent cholangitis, biliary strictures, or segmental hepatic atrophy, due to the increased risk of CCA [3]. Surgical management also offers a lower recurrence rate of stones compared to non-surgical approaches such as endoscopic and percutaneous treatments, with or without lithotripsy, using choledochoscopy [2,4].

Laparoscopic hepatectomy provides comparable outcomes to open surgery with advantages such as less blood loss, faster recovery, and fewer postoperative complications, though it remains technically demanding. Ultimately, the choice of surgical approach should be based on disease extent, anatomical considerations, and the surgeon's expertise [5].

This case report was previously presented as a meeting video at the 2025 Americas Hepato-Pancreato-Biliary Association (AHPBA) meeting, held in Miami, United States, on March 20-23, 2025.

## Case presentation

A 65-year-old male patient presented with a history of abdominal pain without clinical jaundice. The patient was afebrile at the time of evaluation but reported chills. No abdominal tenderness was observed upon physical examination. Laboratory tests revealed mild abnormalities in liver function tests with a cholestatic pattern (Table 1).

**Table 1:** Laboratory tests AST: aspartate aminotransferase; ALT: alanine aminotransferase; GGT, gamma-glutamyl transferase; SGOT: serum glutamic-oxaloacetic transaminase; SGPT: serum glutamic-pyruvic transaminase

Test	Result	Reference range
AST (SGOT)	680 U/L ↑	Up to 50 U/L
ALT (SGPT)	681 U/L ↑	Up to 50 U/L
AST/ALT Ratio	1.0	0.8 - 1.5
GGT	163 U/L ↑	Up to 60 U/L
Alkaline Phosphatase	187 U/L ↑	45 - 115 U/L
Total Bilirubin	1.51 mg/dL ↑	Up to 1.0 mg/dL
Direct Bilirubin	1.41 mg/dL ↑	Up to 0.3 mg/dL
Prothrombin Time	87%	70 - 120 %
Sodium	139 mEq/L	135 - 145 mEq/L
Potassium	4.6 mEq/L	3.5 - 5.0 mEq/L
Chloride	102 mEq/L	100 - 108 mEq/L
Lactic Acid	6.5 mmol/L ↑	0.6 - 1.4 mmol/L
Creatinine	1.03 mg/dL	0.70 - 1.20 mg/dL
Hemoglobin	12.6 g/dL ↓	13.5 - 17.5 g/dL
Hematocrit	35.9% ↓	41.0 - 53.0 %
White Blood Cells	14.7 x10³/µL↑	4.5 - 11.0 x10³/µL
Platelets	255 x10³/µL	140 - 400 x10³/µL

Magnetic resonance cholangiopancreatography (MRCP) was performed, revealing cholelithiasis, choledocholithiasis, and hepatolithiasis in segments V and VIII (Video 1).

**Video 1:** Magnetic resonance imaging (T2-weighted phase) At the beginning of the video, the axial plane is shown, followed by the coronal plane. In both views, the presence of cholelithiasis, choledocholithiasis, and dilatation of the bile ducts in segments V and VIII can be observed, suggestive of hepatolithiasis. Additionally, a notable finding is the gallbladder located to the left of the falciform ligament plane.

**Figure 1:**
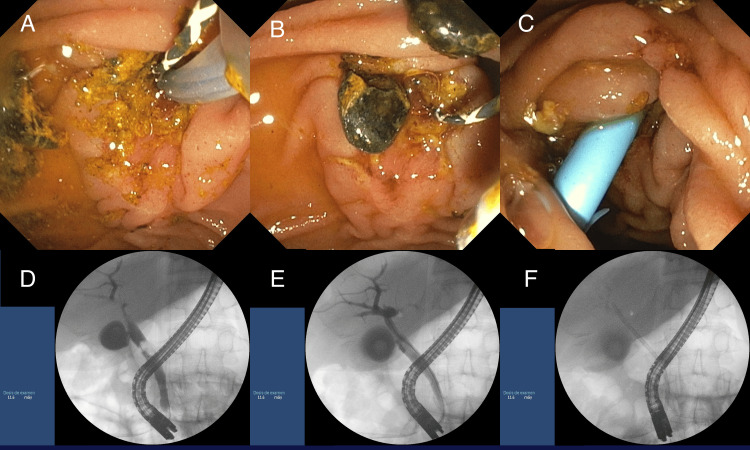
Endoscopic retrograde cholangiopancreatography (ERCP) Papillotomy was performed with the extraction of multiple stones and biliary sludge, and a 10 Fr plastic stent was placed in the right duct. (A) Papillotomy and extraction of biliary sludge; (B) Extraction of choledocholithiasis; (C) A 10 Fr plastic stent was placed in the right duct; (D) Initial cholangiography showing choledocholithiasis; (E) Post-extraction cholangiography confirmed complete clearance of the common bile duct; (F) The recently inserted 10 Fr plastic stent is visible in the right hepatic duct.

The patient was started on antibiotic therapy with ciprofloxacin. In a multidisciplinary meeting, it was decided to perform an ERCP and cholangioscopy for the extraction of the choledocholithiasis. During the procedure, papillotomy was performed with the extraction of multiple stones and biliary sludge, and a 10 Fr plastic stent was placed in the right duct (Figure 1). In addition, a stenosis of the right hepatic duct was found and biopsied. The biopsy result revealed inflammatory tissue, with neoplasia not completely excluded.

The patient had a favorable clinical course, remained afebrile, asymptomatic, and had clear urine. He was presented again in a multidisciplinary meeting, and an elective laparoscopic right hepatectomy was scheduled.

The surgery (Video 2) began with the mobilization of the right hepatic lobe. An accessory venous branch of segment VI was identified and divided with a stapler. The hepatocaval ligament (HCL) was identified, dissected, and divided using a stapler. The right hepatic vein was dissected, and a hanging maneuver was performed. Once this was completed, dissection of the pedicle was initiated. A Hem-o-lok clip was applied at the cystic-gallbladder junction. The right portal vein was identified and marked with a vascular tape. The cholecystectomy was completed. 

**Video 2:** Totally Laparoscopic Right Trisectionectomy

It is important to note that this patient had an anatomical variation, with the gallbladder located to the left of the falciform ligament plane, which altered the visualization of the anatomy and added complexity to the identification of structures. Dissection of the pedicle continued, identifying an arterial branch to segment IV, which was also marked with a vascular tape. The right anterior and posterior hepatic arteries were subsequently identified, and a vascular bulldog clamp was placed. Intraoperative colangiography and ultrasonography confirmed the known hepatolithiasis in segments V and VIII, as well as additional hepatolithiasis in segment IV. Therefore, a decision was made to proceed with an extended right hepatectomy.

**Figure 2:**
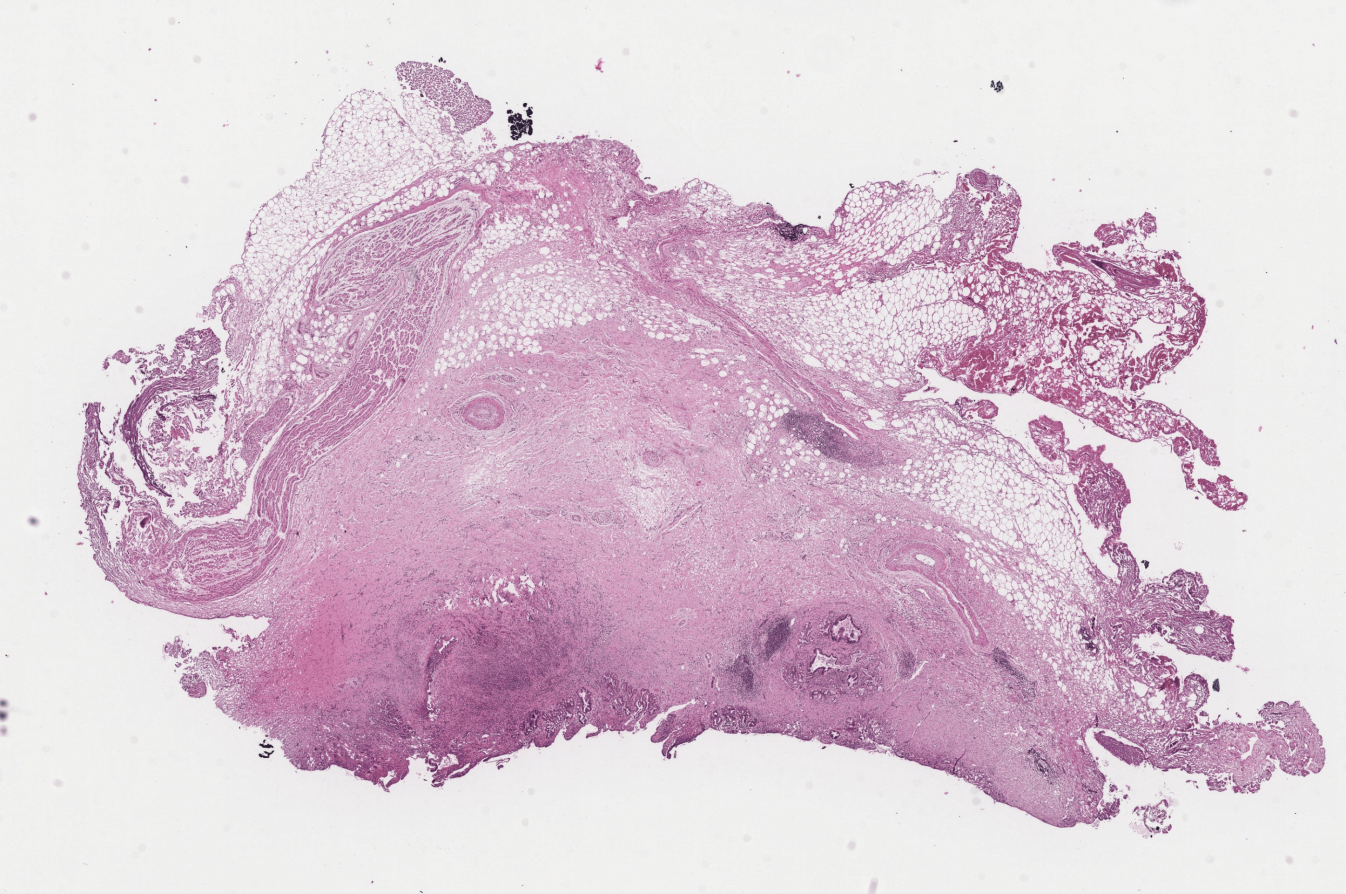
Biopsy of the margin of the right hepatic duct. Biopsy was negative for neoplasia

**Figure 3:**
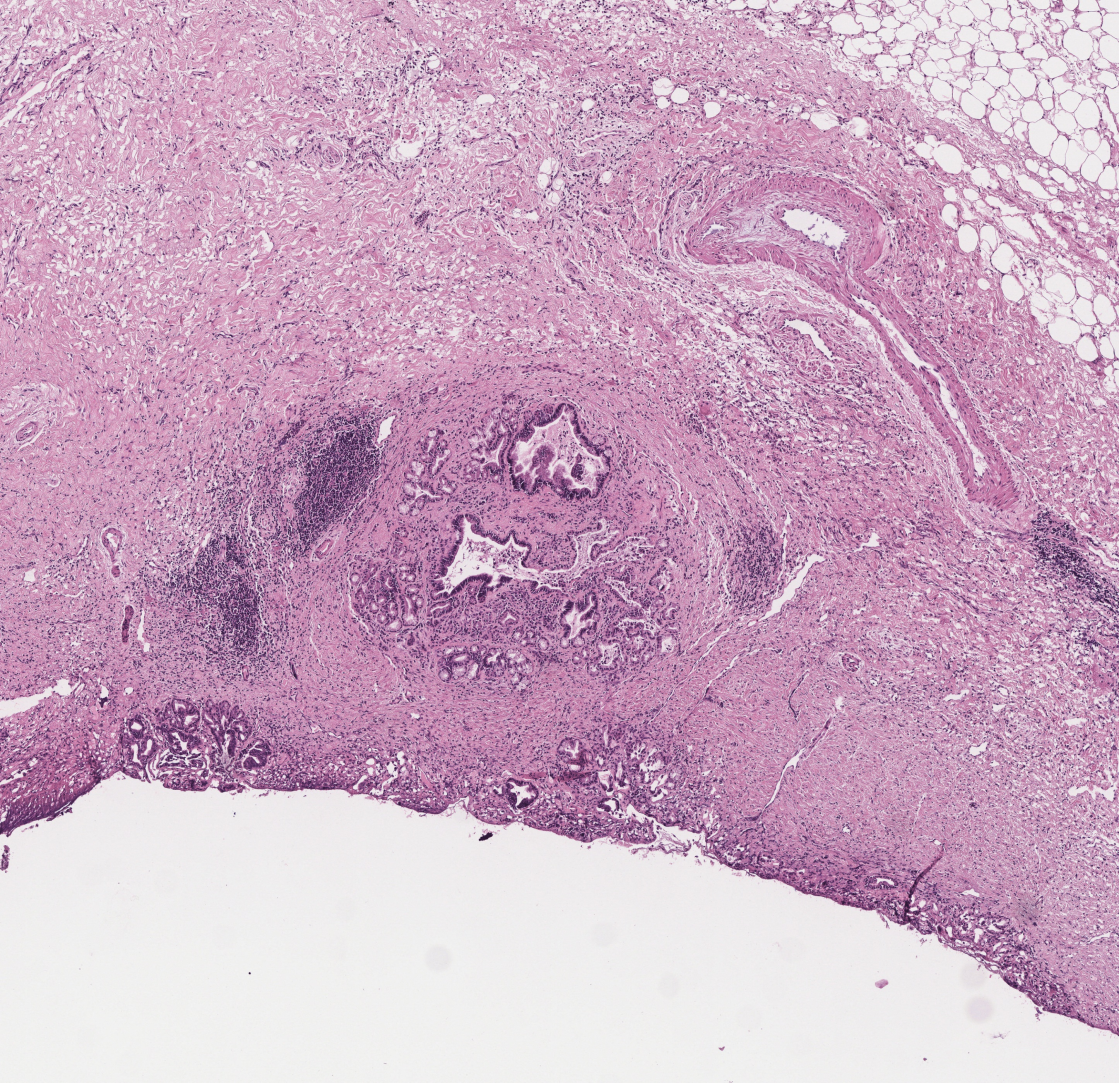
Biopsy of the margin of the right hepatic duct. Magnified view.

**Figure 4:**
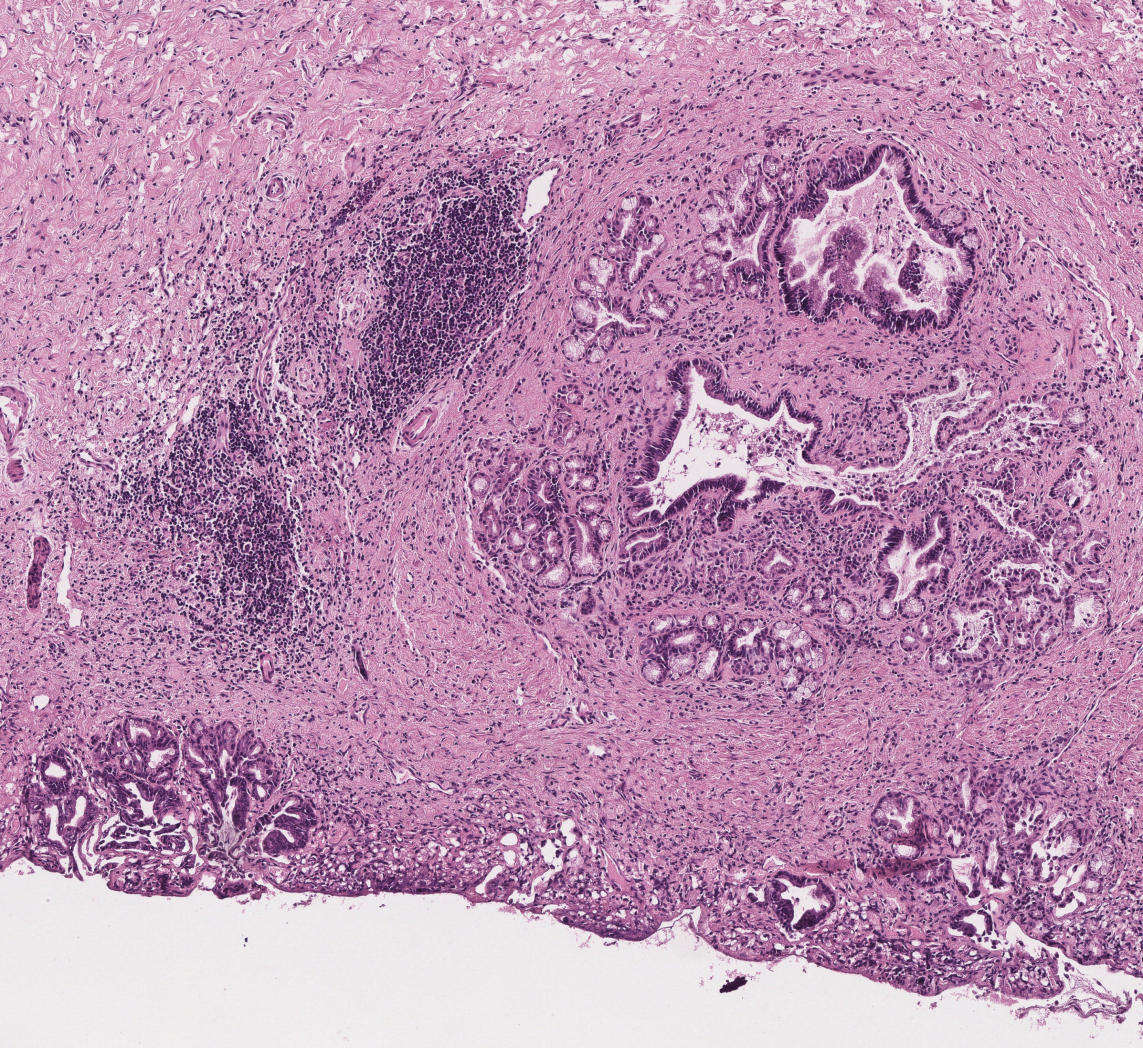
Biopsy of the margin of the right hepatic duct. Magnified view. Biliary duct with foci of chronic inflammation. Slightly hyperchromatic columnar epithelium that mantains lobular architecture.

Parenchymal transection began along the falciform ligament. Inflamed pedicular elements in segments IV due to hepatolithiasis were identified and divided. The middle hepatic vein was identified and transected using a stapler. The portal pedicle to segment IV was divided using a stapler. The right hepatic artery was identified and divided between clips, followed by the right portal vein, which was divided between Hem-o-lok clips. The anterior branch to segment IV was finally transected. The right hepatic duct was divided using scissors, and the stent previously placed during the ERCP was removed. The transection was completed by identifying the anterior and posterior ducts. Hepatic transection was finalized. The right hepatic vein was transected using a stapler. Finally, a right hepatic duct margin was sent for frozen section biopsy, which was negative for neoplasia (Figures 2-4). The procedure was concluded by securing the lateral segments.

## Discussion

Hepatolithiasis is a rare condition in Western countries; however, its diagnosis has increased over the last decade, largely due to the widespread use of advanced imaging technologies such as MRI and MRCP [6].

Multiple treatment modalities exist, with endoscopic and percutaneous methods, sometimes combined with lithotripsy, being the most commonly employed. However, these approaches are associated with high recurrence rates, especially when underlying biliary strictures are not addressed, as they are the main cause of recurrence. Although these techniques can offer high short-term success rates (up to 96% in stone clearance), recurrence rates can reach between 30% and 54%. In this context, hepatic resection remains the most effective option for long-term cure, as it allows for the removal of stones, strictures, and atrophic or damaged liver parenchyma, while also reducing the risk of CCA [6,7].

In Jarufe et al.'s series, surgery cleared bile duct stones in over 91% of patients, and this increased to 98% with further procedures. Recurrence was low (13.5% over five years) and usually manageable with ERCP, supporting liver resection as a strong option in selected cases [1]. García et al. reported a 30.8% complication rate, mostly infections in patients with prior cholangitis [7]. Bile leaks were the most frequent issue after major resections, but there were no deaths or cases of liver failure.

Totally laparoscopic right hepatectomy has gained traction in recent years as a treatment for hepatolithiasis, although it remains technically demanding. This is primarily due to chronic inflammatory changes associated with biliary pathology, such as adhesions and anatomical distortions, which can alter normal tissue planes, particularly around the hepatic hilum, making dissection more complex and increasing the risk of intraoperative bleeding. For this reason, precise control of vascular and biliary structures, along with the use of advanced hemostatic techniques, is essential. In this context, intraoperative cholangiography or indocyanine green fluorescence imaging can be a crucial tool for accurate anatomical identification. Given these challenges, this procedure should be performed by surgical teams with high levels of expertise and experience in laparoscopic liver surgery.

As illustrated in Video 2, the primary technical difficulties encountered during the procedure were related to localized inflammation, particularly around the pedicle and segment IV, and also due to the fact that the patient had an anatomical variation, with the gallbladder located to the left of the falciform ligament plane, which altered anatomical visualization and affected tissue planes making the identification of key anatomical structures more challenging than usual. Dissection of vascular elements, including the right anterior and posterior hepatic arteries and the portal pedicles, required special attention due to their close relation to inflamed areas. Additionally, the presence of hepatolithiasis in segments V, VIII, and IV added another layer of difficulty, especially during biliary dissection. These factors collectively contributed to the complexity of the extended right hepatectomy.

The management of bilateral hepatolithiasis still remains controversial, as it is associated with higher recurrence rates, longer hospital stays, and increased need for biliary-enteric reconstructions [6,7].

The association between hepatolithiasis and CCA is well documented, with reported incidences ranging from 2% to 23%. Chronic inflammation due to persistent biliary stasis and recurrent stone disease is a known risk factor [6,7]. However, it's important to note that the risk of developing CCA may persist even after liver resection [3]. Therefore, appropriate follow-up and encouragement of a healthy lifestyle, while limiting risk factors, are recommended.

## Conclusions

Laparoscopic extended right hepatectomy is a feasible and effective surgical option for the management of complex hepatolithiasis, particularly in cases involving biliary strictures and segmental disease. In this case, the minimally invasive approach allowed for complete resection of affected segments, resolution of biliary obstruction, and removal of all identified hepatolithiasis, with no intraoperative complications or evidence of malignancy.

Given the technical complexity associated with inflammation and distorted anatomy, these procedures should be reserved for specialized centers with expertise in advanced hepatobiliary surgery. Surgical resection offers the benefit of long-term stone clearance and reduced risk of recurrence or malignant transformation, emphasizing its value in selected patients with localized hepatolithiasis.
